# Detecting disease-associated genotype patterns

**DOI:** 10.1186/1471-2105-10-S1-S75

**Published:** 2009-01-30

**Authors:** Quan Long, Qingrun Zhang, Jurg Ott

**Affiliations:** 1 Beijing Institute of Genomics, Chinese Academy of Sciences, No. 7 Bei Tu Cheng West Road, Beijing 100029, PR China; 2 Current address: Wellcome Trust Sanger Institute, Cambridge, UK

## Abstract

**Background:**

In addition to single-locus (main) effects of disease variants, there is a growing consensus that gene-gene and gene-environment interactions may play important roles in disease etiology. However, for the very large numbers of genetic markers currently in use, it has proven difficult to develop suitable and efficient approaches for detecting effects other than main effects due to single variants.

**Results:**

We developed a method for jointly detecting disease-causing single-locus effects and gene-gene interactions. Our method is based on finding differences of genotype pattern frequencies between case and control individuals. Those single-nucleotide polymorphism markers with largest single-locus association test statistics are included in a pattern. For a logistic regression model comprising three disease variants exerting main and epistatic interaction effects, we demonstrate that our method is vastly superior to the traditional approach of looking for single-locus effects. In addition, our method is suitable for estimating the number of disease variants in a dataset. We successfully apply our approach to data on Parkinson Disease and heroin addiction.

**Conclusion:**

Our approach is suitable and powerful for detecting disease susceptibility variants with potentially small main effects and strong interaction effects. It can be applied to large numbers of genetic markers.

## Background

It is now generally accepted that complex genetic traits such as diabetes and schizophrenia are under the influence of multiple interacting loci and environmental triggers, each with a possibly small effect. Thus, to overcome the limitations of traditional single-locus association analysis (looking for main effects of single marker loci), various methods have been developed to investigate the joint disease association of multiple markers. As an approximation to multivariate analysis [1], sums of single-locus association statistics [2] have proven to be efficient, powerful [3], and applicable to large numbers of markers. A more direct approach is to investigate whether sets of specific alleles or genotypes at different loci occur more frequently in case than control individuals [4,5].

Haplotypes are sets of alleles, one each at different loci, on a chromosome. Each individual has two haplotypes but, because of unknown phase, it is generally not possible to identify the two specific haplotypes in a given individual. For this reason, we prefer to work with sets of *geno*types at different loci (diplotypes). To allow for possible interactions between any genes, we consider genotypes at different loci, wherever these occur in the genome, and refer to such sets of genotypes as *genotype patterns*.

The simplest situation to consider is that of two genetic markers. Throughout this paper we will focus on single nucleotide polymorphisms (SNPs). One pair of SNPs, each with 3 genotypes, comprises a maximum of 9 genotype patterns. A possible strategy is to investigate all pairs of SNPs to see for each pair whether the genotype pattern frequencies are different in case and control individuals [6]. Several diseases have been described requiring a mutation at two different loci while occurrence of a mutation at only one locus does not lead to disease [7].

At least in experimental organisms, researchers have developed methods to specifically search for epistatic interactions between two loci, with the aim to detect networks of interacting loci [8]. In human genetics, several approaches to detecting the joint effects of multiple susceptibility variants have been proposed [9-14]. However, many of these methods are applicable only to small numbers of SNPs and are not suitable for genome-wide analysis of 100,000s of markers. For this reason, two-step approaches are more promising [2,15,16]. Specifically, for two-locus disease models, a two-step approach has been proposed that initially selects SNPs based on significant single-locus tests, with logistic regression analysis of all possible main and interaction effects at the second stage [16]. Here, we proceed in a similar manner but with important differences: initial selection of SNPs is also carried out based on their single-locus test results but irrespective of whether these are significant, and the number of these test SNPs to be carried forward for second-stage analysis may be varied from 1 up to any desired maximum number, *m*, limited only by computer resources. Instead of logistic regression analysis, we propose to test whether for a given number *m *of test SNPs the frequencies of genotype patterns is different in case and control individuals.

It should be noted that genotype pattern frequencies comprise main effects (at one or multiple loci) *and *epistatic interaction effects. Here our focus is on genotype patterns with an interest in finding pattern frequencies reflecting a high degree of epistatic interaction so that disease-associated SNPs may be found even though they contribute to disease more through interaction effects than direct main effects. Such genes have been found, for example, in barley [17], and so-called digenic traits are known in humans [7].

## Methods

### Pattern recognition approach

Consider a dataset with a certain number of case and control individuals, each genotyped at a number *M *of SNPs, where *M *might be equal to 1 million, say. We want to find disease-associated patterns of *m *SNPs, *m *<<*M*, each pattern consisting of a genotype at each of the *m *SNPs. The total number of subsets of *m *SNPs that can be formed from *M *SNPs is equal to *M*!/[*m*!(*M *- *m*)!], and each of these subsets contains a maximum of 3^m ^genotype patterns. Thus, searching through all possible patterns of even a small number *m *of SNPs is an enormous task and generally too computationally intensive. One might consider picking a random sample of all possible genotype patterns and evaluate them for association with disease, that is, testing whether pattern frequencies are significantly different between case and control individuals. A more satisfactory solution seems to select patterns with certain desirable properties, for example, patterns formed of SNPs that have been suitably prioritized [18]. In the absence of biological and sequence-based information, the statistically most relevant prioritization is based on the significance level (*p*-value) achieved in an association test. In other words, we select SNPs having the largest main effects. Presumably, epistatic interaction as a disease causing mechanism will also result in some main effects although these are not necessarily significant. Such main effects are at least predicted under logistic regression models (see below). We propose the following two-step approach.

#### Pattern test

(1) For each of a possibly very large number *M *of SNPs, an association test is carried out, for example, chi-square is computed for a 2 × 3 contingency table of genotypes, where the two rows correspond to cases and controls, and columns refer to the three SNP genotypes. The top *m *SNPs (with largest chi-square) are singled out for further analysis, where *m *may be guided by considerations such as potential importance for disease of the genes containing these SNPs. (2) For the selected *m *test SNPs, all genotype patterns are identified and their frequencies established. Those patterns with frequencies below 5% in cases and controls each are pooled into a "rare" class. For example, with *m *= 3, the total number of genotype patterns is 27. Some patterns may not occur in the given data and rare patterns are pooled into a single class so that only *r *patterns with appreciable frequency are observed. These data are now arranged in a 2 × *r *contingency table, for which chi-square and an associated significance level, *p*_m_, is computed.

We take *p*_m _as our experiment-wise test statistic (rather than chi-square, which may have different degrees of freedom depending on the number *s *of genotype patterns). Clearly, *p*_m _may not be taken at face value as a significance level – the type 1 error would be grossly inflated because *p*_m _is based on the ascertainment of the *m *best SNPs. Thus, we need to establish the significance level associated with our observed value of *p*_m_, that is, determine the probability of obtaining a value as small or smaller than the one observed without there being an association (null hypothesis). We do this in *N *randomization samples, that is, we randomly permute the labels "case" and "control" and leave everything else intact. In each randomization sample, the top *m *SNPs are determined, wherever they occur in the genome, and *p'*_m _is computed in analogy to *p*_m _in the observed dataset. A large number of randomization samples will then furnish an approximate null distribution for *p*_m_, from which the significance level is obtained as the proportion of randomization samples with *p'*_m _= *p*_m_. Unless noted otherwise, our calculations are carried out with *N *= 50,000 randomization samples. We compare our pattern test with the following conventional SNP-by-SNP approach.

#### Single-locus test

For each of the *m *best SNPs we compute chi-square for a 2 × 3 contingency table, with rows corresponding to cases/controls and columns representing the three SNP genotypes. Significance levels associated with each of the *m *SNPs are again evaluated in randomization samples. That is, for each observed chi-square the proportion of randomization samples is determined in which a chi-square as large or larger than the one observed occurs. In other words, the reference (null) distribution is that of the largest chi-square [1].

### Disease model and power calculations

To evaluate whether our pattern test does better than the single-locus test, we carry out power calculations by generating case and control individuals under a suitable disease inheritance model. Because of its flexibility, we apply a logistic regression model [19] as follows. We assume *m*_d _= 3 disease loci, each with two equally frequent alleles *A *and *B*. At the *i*-th disease locus, we assign a code *x*_i _= -1 to genotypes *AA *and *AB*, and a code *x*_i _= +1 to genotype *BB*. Thus, genotype codes of -1 and +1 have associated respective probabilities of occurrence of 0.75 and 0.25. Then the logistic disease model is given by

log [*f*/(1 - *f*)] = *a*_0 _+ *a*_1_(*x*_1 _+ *x*_2 _+ *x*_3_) + *a*_2_(*x*_1_*x*_2 _+ *x*_1_*x*_3 _+ *x*_2_*x*_3_) + *a*_3_*x*_1_*x*_2_*x*_3 _= *s*,

where *f *is the conditional probability of being affected given the genotype pattern specified by the *x*_i_'s (*f *is often called penetrance) and the *a*_i _are coefficients representing main (*a*_1_) and interaction effects (*a*_2_, *a*_3_). For simplicity, all main effects are the same and all first-order (pairwise) interaction effects are the same, while *a*_1 _= *a*_2 _= *a*_3 _= 0 corresponds to the null hypothesis of no genetic effects. For any setting of *a*_i _> 0 (*i *> 0), the parameter *a*_0 _is chosen in such a way that the model predicts a disease prevalence of *K *= 0.05. For example, this prevalence is predicted by parameter values *a*_1 _= *a*_2 _= 0, *a*_3 _= 3, and *a*_0 _= -5.05. Note that such a model containing only a second-order interaction effect does induce some direct genotype effects at each of the three disease loci. Depending on the genotype pattern, these parameter settings lead to odds ratios (ORs) for disease ranging from 0.0034 through 3.12 and, at each single locus, lead to ORs of 0.58 and 1.71 for the respective genotype codes *x*_i _= -1 and *x*_i _= +1.

Initially, we only consider *m*_d _= 3 SNPs in complete association with disease susceptibility variants. This way we directly compare power between the pattern and single-locus tests. Subsequently we also consider the effects of unassociated SNPs and how they degrade power.

## Results

### Simulation with disease SNPs

**Figure 1:**
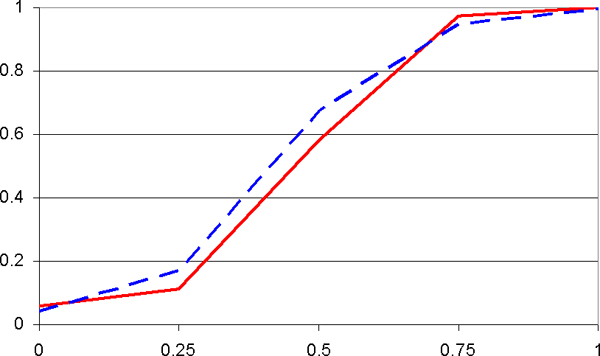
**Power (*Y*-axis) as a function of main effects (*x*-axis)**. For a pure main-effects model (*x*-axis = *a*_1 _> 0; interaction effects *a*_2 _= *a*_3 _= 0 are all zero), Figure 1 shows that pattern (solid red line) and single-locus (broken blue line) approaches have virtually the same power.

**Figure 2:**
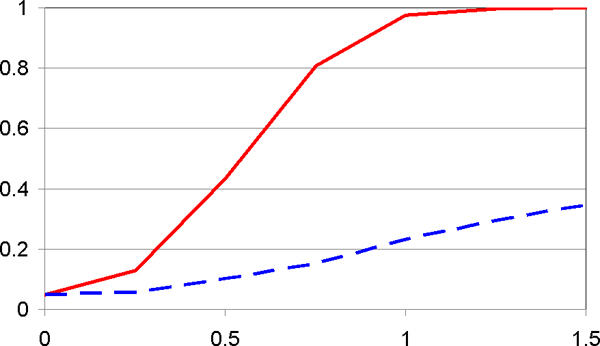
**Power (*y*-axis) as a function of interaction effects (*x*-axis)**. With strong second order interaction effects (x-axis = *a*_3_; *a*_1 _= *a*_2 _= 0), the pattern approach (solid red line) is much more powerful than the single-locus test (broken blue line).

Power calculations in this section were carried out for sample sizes of 50 case and control individuals each. For a pure main-effects model (*a*_1 _> 0, *a*_2 _= *a*_3 _= 0), Figure 1 shows that pattern and single-locus approaches have virtually the same power. This is to be expected: Disease SNPs are assumed uncorrelated and each acts independently. On the other hand, in the presence of second-order interaction effects (*a*_1 _= *a*_2 _= 0, *a*_3 _> 0), with direct single-locus effects only present inasmuch as they are induced by interaction effects (see above), Figure 2 demonstrates the tremendous power advantage of the pattern approach over the single-locus approach.

### Simulation with disease and unassociated SNPs

With *m*_d _susceptibility SNPs and *M *unassociated SNPs (total of *m*_d _+ *M *SNPs), we need to define "success" in our simulations. In a given randomization sample, it is no longer sufficient that *p'*_m _= *p*_m_. In addition, we require that the particular subset of *m *= 3 ascertained SNPs with highest chi-square values comprise at least one disease SNP. An analogous rule applies to the single-locus test.

**Figure 3:**
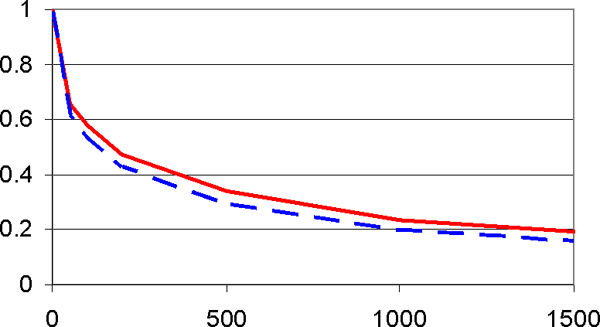
**Power (*y*-axis) as a function of the total number, *M*, of SNPs**. Figure 3 shows that power is strongly reduced by the presence of random (unassociated) markers. For 100 cases and controls each, under a model with main and interaction effects (*a*_1 _= 1, *a*_2 _= 0, *a*_3 _= 6), pattern approach (solid red line) and single-locus approach (broken blue line) suffer approximately the same power losses.

**Figure 4:**
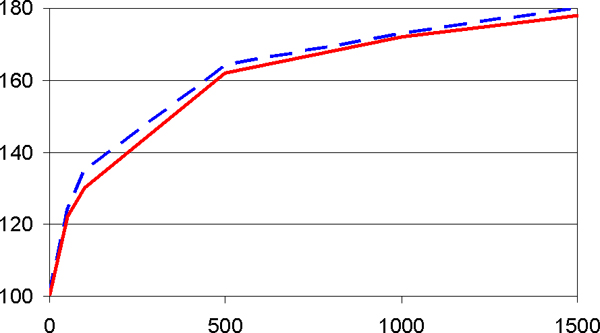
**Sample sizes (numbers of cases and controls, *y*-axis) for power of 80%**. The power loss due to increasing numbers *M *of unassociated SNPs may be offset by increased sample sizes. Figure 4 shows the numbers of cases and controls each (*y*-axis) that are necessary to maintain power of 80% in the presence of *M *of unassociated SNPs (*x*-axis). Parameter values are as in Figure 3. Pattern approach (solid red line) and single-locus approach (broken blue line) furnish virtually identical results.

For 100 case and controls each, with *a*_1 _= 1, *a*_2 _= 0, and *a*_3 _= 6, Figure 3 shows the deleterious effects of "noise" SNPs. In the presence of *M *= 1500 unassociated SNPs, power drops from 100% to 20%, where power loss is somewhat smaller for the pattern than the single-locus approach although both suffer greatly. This power loss may be offset by using additional observations. With the same parameter values, to maintain a power of 80%, Figure 4 shows that for the pattern method an increase in sample size from 100 to 180 is sufficient to offset the harmful effects of "noise" SNPs. As the number of unassociated SNPs increases, required sample sizes initially increase strongly but later less so. The required sample size increase is virtually the same for the two association tests considered here so that only one curve is shown in Figure 4.

### Choice of number Of SNPs for analysis

**Figure 5:**
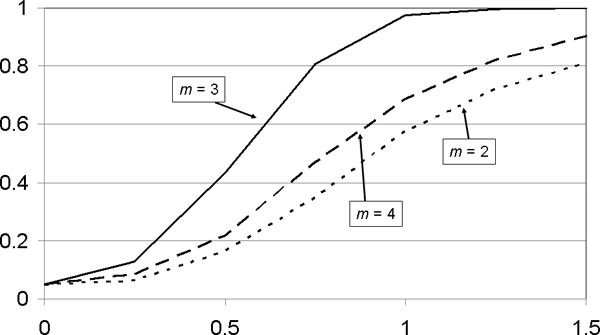
**Power (*y*-axis) of interaction effects (*x*-axis) for different numbers of test SNPs**. For an epistatic disease model (*a*_1 _= *a*_2 _= 0, *a*_3 _> 0) with three disease SNPs, *m*_d _= 3 (no other SNPs, 100 cases and controls each), power (*y*-axis) is displayed as a function of the interaction coefficient, *a*_3 _(*x*-axis), for different numbers *m *of test SNPs. Power is highest when *m *= *m*_d_.

So far, we analyzed data looking for patterns of length *m *= 3 while the data were generated with *m*_d _= 3. In reality, of course, the number *m*_d _of disease variants is unknown. Thus, for our epistatic disease model (*a*_1 _= *a*_2 _= 0, *a*_3 _> 0, *m*_d _= 3, no other SNPs, 100 cases and controls each), we estimated power for different numbers *m *of best SNPs considered. Not unexpectedly, power is highest when *m *= *m*_d _(Figure 5). Also, power is better when *m *> *m*_d _then when *m *<*m*_d_. As highest power is expected to translate into smallest *p*-value, trying different numbers *m *of SNPs is expected to estimate the number of disease variants in a dataset.

### Analysis of Parkinson's Disease dataset

A dataset with approximately 540 case and control individuals and approximately 408,000 SNPs genome-wide is available for re-analysis [20]. For our purpose, we chose to work with the 19,494 SNPs on chromosome 11 that passed our quality control measures; SNP rs10501570 on that chromosome was previously associated with Parkinson's Disease (PD) [20]. We decided to work with a subset size of *m *= 3 SNPs. In chi-square tests for 2 × 3 tables (case/control phenotype versus three SNP genotypes), the three largest values occurred for SNPs rs12364577, rs1377470, and rs10501570. In 10,000 randomization samples, the largest chi-square had an associated significance level of 5/10,000 = 0.0005 (corrected for multiple testing) while the other two chi-square values had associated *p*-values of 0.0023 and 0.0030. Forming genotype patterns for these three SNPs, we found five patterns with frequencies of at least 0.05 in either case or control individuals; the remaining patterns were combined into a "rare" class. The chi-square for the resulting 2 × 6 table had an associated randomized significance level of 0.0064. Evidently, for this dataset the single-locus test was more effective than our pattern test (0.0005 < 0.0064), presumably because of strong single-locus effects.

### Analysis of a dataset for heroin addiction

In 104 former severe heroin addicts and 101 control individuals (all Caucasians), a case-control association analysis was carried with 10,000 SNPs [21]. Single-locus analysis furnished nonsignificant results: The largest chi-square in 2 × 3 tables of genotypes had an associated *p*-value of 0.15 (obtained in 10,000 randomization samples). On the other hand, evaluating genotype patterns for the three SNPs with individually smallest *p*-values revealed five common patterns, and the resulting 2 × 6 table of pattern frequencies had an associated experiment-wise significance level of 0.026. As reported in [21], a specific genotype pattern was associated with heroin addiction with an odds ratio of 6.25 while none of the component SNPs by themselves were significantly disease-associated. Interestingly, when we investigated genotype patterns comprising two, four, or five SNPs, the significance levels associated with the corresponding pattern tables were all in excess of 0.30. Thus, the specific set of three genetic variants identified seems to predispose to disease [21].

## Discussion

We report here a new multi-locus method for genetic case-control association analysis that jointly evaluates main and interaction effects of multiple genetic variants. In contrast to our *Set Association *method developed previously [2], it is not based on main effects of individuals SNPs only. Our method is able to find epistatic interaction effects although the test SNPs chosen for analysis are determined by SNP-specific association statistics. Clearly, if SNPs have weak individual effects then they are unlikely to be picked up by our approach. In an application to a study of former heroin addicts, our genotype pattern method clearly showed its advantages over standard single-locus methods.

We implemented our approach in a computer program, *RandomPat*, which is available on request and will soon be available for downloading from our website [22].

In its current implementation, our method has a shortcoming in that it cannot handle missing observations. Thus, for *m *SNPs it will ignore patterns that contain one or more missing genotypes.

A major advantage of our approach is that the number, *m*, of test SNPs can be chosen by the investigator. Varying *m *from 1 up to a suitable upper limit will show, which number of test SNPs is most significant in a given dataset. As demonstrated in the previous section, such an approach is expected to estimate the number of disease variants if they are all of equal strengths.

## Conclusion

As we demonstrated, our method is clearly superior to the traditional approach of looking for single-locus effects. In addition, it is suitable for estimating the number of disease variants in a dataset. On the other hand, purely epistatic loci (no main effects by themselves) cannot be detected by our approach.

## Competing interests

The authors declare that they have no competing interests.

## Authors' contributions

Quan Long contributed to the concept of our approach and wrote the computer program. Qingrun Zhang participated in the development of the method and contributed to programming. Jurg Ott participated in methods development and wrote the manuscript.

## References

[B1] Manly BFJ (2007). Randomization, bootstrap, and Monte Carlo methods in biology.

[B2] Hoh J, Wille A, Ott J (2001). Trimming, weighting, and grouping SNPs in human case-control association studies. Genome Res.

[B3] Kim S, Zhang K, Sun F (2003). Detecting susceptibility genes in case-control studies using set association. BMC Genet.

[B4] Ritchie MD, Hahn LW, Roodi N, Bailey LR, Dupont WD, Parl FF, Moore JH (2001). Multifactor-dimensionality reduction reveals high-order interactions among estrogen-metabolism genes in sporadic breast cancer. Am J Hum Genet.

[B5] Moore JH, Ritchie MD (2004). STUDENTJAMA. The challenges of whole-genome approaches to common diseases. Jama.

[B6] Hoh J, Ott J (2003). Mathematical multi-locus approaches to localizing complex human trait genes. Nat Rev Genet.

[B7] Ming JE, Muenke M (2002). Multiple hits during early embryonic development: digenic diseases and holoprosencephaly. Am J Hum Genet.

[B8] Roguev A, Wiren M, Weissman JS, Krogan NJ (2007). High-throughput genetic interaction mapping in the fission yeast Schizosaccharomyces pombe. Nat Methods.

[B9] Nelson MR, Kardia SL, Ferrell RE, Sing CF (2001). A combinatorial partitioning method to identify multilocus genotypic partitions that predict quantitative trait variation. Genome Res.

[B10] Zee RY, Hoh J, Cheng S, Reynolds R, Grow MA, Silbergleit A, Walker K, Steiner L, Zangenberg G, Fernandez-Ortiz A (2002). Multi-locus interactions predict risk for post-PTCA restenosis: an approach to the genetic analysis of common complex disease. Pharmacogenomics J.

[B11] Ruczinski I, Kooperberg C, LeBlanc ML (2004). Exploring interactions in high-dimensional genomicdata: an overview of LogicRegression, with applications. Journal of Multivariate Analysis.

[B12] Kooperberg C, Ruczinski I (2005). Identifying interacting SNPs using Monte Carlo logic regression. Genet Epidemiol.

[B13] Lauer MS, Alexe S, Pothier Snader CE, Blackstone EH, Ishwaran H, Hammer PL (2002). Use of the logical analysis of data method for assessing long-term mortality risk after exercise electrocardiography. Circulation.

[B14] Reddy A, Wang H, Yu H, Bonates TO, Gulabani V, Azok J, Hoehn G, Hammer PL, Baird AE, Li KC (2008). Logical Analysis of Data (LAD) model for the early diagnosis of acute ischemic stroke. BMC Med Inform Decis Mak.

[B15] Hoh J, Wille A, Zee R, Cheng S, Reynolds R, Lindpaintner K, Ott J (2000). Selecting SNPs in two-stage analysis of disease association data: a model-free approach. Ann Hum Genet.

[B16] Marchini J, Donnelly P, Cardon LR (2005). Genome-wide strategies for detecting multiple loci that influence complex diseases. Nat Genet.

[B17] Xu S, Jia Z (2007). Genomewide analysis of epistatic effects for quantitative traits in barley. Genetics.

[B18] Jiang R, Yang H, Zhou L, Kuo CC, Sun F, Chen T (2007). Sequence-based prioritization of nonsynonymous single-nucleotide polymorphisms for the study of disease mutations. Am J Hum Genet.

[B19] Xu S (2007). An empirical Bayes method for estimating epistatic effects of quantitative trait loci. Biometrics.

[B20] Fung HC, Scholz S, Matarin M, Simon-Sanchez J, Hernandez D, Britton A, Gibbs JR, Langefeld C, Stiegert ML, Schymick J (2006). Genome-wide genotyping in Parkinson's disease and neurologically normal controls: first stage analysis and public release of data. Lancet Neurol.

[B21] Nielsen DA, Ji F, Yuferov V, Ho A, Chen A, Levran O, Ott J, Kreek MJ (2008). Genotype patterns that contribute to increased risk for or protection from developing heroin addiction. Mol Psychiatry.

[B22] Statistical Genetics Beijing. http://www.genemapping.cn.

[B23] Coriell NINDS Collection. http://ccr.coriell.org/ninds.

